# PSMA-PET follow-up to assess response in patients not receiving PSMA therapy: Is there value beyond localization of disease?

**DOI:** 10.7150/thno.96738

**Published:** 2024-06-11

**Authors:** Alina T. Küper, David Kersting, Tugce Telli, Ken Herrmann, Axel Rominger, Ali Afshar-Oromieh, Leonor Lopes, Sofia Karkampouna, Kuangyu Shi, Moon Kim, Boris Hadaschik, Christopher Darr, Lale Umutlu, Wolfgang P. Fendler, Robert Seifert

**Affiliations:** 1 Department of Nuclear Medicine and German Cancer Consortium (DKTK), University Hospital Essen, University of Duisburg-Essen, Essen, Germany.; 2 Department of Nuclear Medicine, University Hospital Bern, University of Bern, Bern, Switzerland.; 3 Graduate School for Cellular and Biomedical Sciences, University of Bern, Bern, Switzerland.; 4 Urology Research Laboratory, Department for BioMedical Research, University of Bern, 3008, Bern, Switzerland.; 5 Department of Urology, Inselspital, Bern University Hospital, University of Bern, Switzerland.; 6 Institute for Artificial Intelligence in Medicine, University Hospital Essen, Essen, Germany.; 7 Department of Urology and German Cancer Consortium (DKTK), University Hospital Essen, University of Duisburg-Essen, Essen, Germany.; 8 Institute of Interventional and Diagnostic and Interventional Radiology and Neuroradiology, University Hospital Essen, Essen, Germany.

**Keywords:** Prostate cancer, PSMA PET, Tumor volume, Bone tumor volume, miTNM

## Abstract

**Introduction:** Prostate Specific Membrane Antigen Positron Emission Tomography (PSMA-PET) is routinely used for the staging of patients with prostate cancer, but data on response assessment are sparse and primarily stem from metastatic castration-resistant prostate cancer (mCRPC) patients treated with PSMA radioligand therapy. Still, follow-up PSMA-PET is employed in earlier disease stages in case of clinical suspicion of disease persistence, recurrence or progression to decide if localized or systemic treatment is indicated. Therefore, the prognostic value of PSMA-PET derived tumor volumes in earlier disease stages (i.e., hormone-sensitive prostate cancer (HSPC) and non-[^177^Lu]Lu-PSMA-617 (LuPSMA) therapy castration resistant prostate cancer (CRPC)) are evaluated in this manuscript.

**Methods:** A total number of 73 patients (6 primary staging, 42 HSPC, 25 CRPC) underwent two (i.e., baseline and follow-up, median interval: 379 days) whole-body [^68^Ga]Ga-PSMA-11 PET/CT scans between Nov 2014 and Dec 2018. Analysis was restricted to non-LuPSMA therapy patients. PSMA-PETs were retrospectively analyzed and primary tumor, lymph node-, visceral-, and bone metastases were segmented. Body weight-adjusted organ-specific and total tumor volumes (PSMAvol: sum of PET volumes of all lesions) were measured for baseline and follow-up. PSMAvol response was calculated as the absolute difference of whole-body tumor volumes. High metastatic burden (>5 metastases), RECIP 1.0 and PSMA-PET Progression Criteria (PPP) were determined. Survival data were sourced from the cancer registry.

**Results:** The average number of tumor lesions per patient on the initial PET examination was 10.3 (SD 28.4). At baseline, PSMAvol was strongly associated with OS (HR 3.92, p <0.001; n = 73). Likewise, response in PSMAvol was significantly associated with OS (HR 10.48, p < 0.005; n = 73). PPP achieved significance as well (HR 2.19, p <0.05, n = 73). Patients with hormone sensitive disease and poor PSMAvol response (upper quartile of PSMAvol change) in follow-up had shorter outcome (p < 0.05; n = 42). PSMAvol in bones was the most relevant parameter for OS prognostication at baseline and for response assessment (HR 31.11 p < 0.001; HR 32.27, p < 0.001; n = 73).

**Conclusion:** PPP and response in PSMAvol were significantly associated with OS in the present heterogeneous cohort. Bone tumor volume was the relevant miTNM region for OS prognostication. Future prospective evaluation of the performance of organ specific PSMAvol in more homogeneous cohorts seems warranted.

## Introduction

In the evolving landscape of prostate cancer (PCa) management, treatment decisions are dependent on the risk of disease progression and, ultimately, their impact on OS 1. Advocating for enhanced treatment response tracking, a European consensus committee, comprising members of EANM and EAU, has recommended the utilization of Prostate Specific Membrane Antigen Positron Emission Tomography/Computer Tomography (PSMA-PET/CT) since 2020 2. PSMA-PET has been widely used for localization of disease, but limited evidence is present on whether it should be used for response assessment. Especially given the ease and low cost of PSA levels, the routine use of PSMA-PET is questioned. However, because of the wide use of PSMA-PET in biochemical recurrent cancer, longitudinal PSMA-PET data is becoming more and more available. Still, in the clinical routine, the reason for its initiation is often not to assess response to therapy, but because clinical features such as PSA indicate tumor progression.

For PSMA radioligand therapy patients, PSMA-PET has been established for response assessment. For example, frameworks like Response Evaluation Criteria in PSMA-imaging v1.0 (RECIP 1.0) have been introduced 3. Yet most clinical decisions are made in earlier disease states for which RECIP 1.0 was not designed 1,4. In addition, semi- to fully-automated segmentation tools can facilitate an innovative and accessible whole-body assessment of PSMA-PET-based total tumor volume (PSMAvol) as a quantitative parameter 5,6. Recently, the PROMISE framework V2.0 has been introduced, which recommends the use of organ specific tumor volume as an exploratory endpoint to assess the risk of treatment failure and monitor response 7. However, only preliminary evidence is available for RECIP 1.0 and novel PSMA-PET-based metrics like organ-specific PSMA tumor volume (PSMAorgan score) have not systematically been used for longitudinal assessment to date 8. Therefore, the aim of this study was to evaluate PSMAvol and organ-specific PSMA-PET-derived tumor volumes for response assessment to prognosticate the outcome of patients with HSPC (hormone sensitive prostate cancer) and CRPC (castration resistant prostate cancer). Parameters were compared to other response metrics in a non ^177^Lutetium-PSMA (LuPSMA) therapy setting to closely reflect real-world clinical scenarios.

## Material and Methods

### Patient cohort

We retrospectively screened our database for patients who had not received Lu-PSMA therapy and underwent at least two (i.e., baseline and follow-up) whole-body [^68^Ga]Ga-PSMA-11 PET/CT scans between November 2014 and December 2018, in the Department of Nuclear Medicine, University Hospital Essen, Germany. PSMA-PET/CT staging was conducted as part of standard clinical workup across various stages and conditions of prostate cancer, including high-risk initial staging, biochemical recurrence, and re-staging/response assessment in castration-sensitive metastatic disease and castration-resistant prostate cancer. There was no standardized interval between the PSMA-PET exams. In case of multiple (≥3) available examinations, the first two scans in the sampling period were chosen. Overall survival was the primary endpoint of the study. Survival data was sourced from the West German Cancer Center's follow-up center, utilizing records from the citizen registration office. The local ethics committee approved the study and waived the need for study specific consent due to its retrospective nature (Ethics committee of the University Hospital Essen, 23-11462-BO).

### PSMA-PET imaging acquisition

PET/CT images were acquired on a Biograph mCT system (Siemens Healthineers, Erlangen, Germany). Before the PET acquisition, a contrast-enhanced whole-body CT scan was performed if not clinically available within 4 weeks before the examination date; otherwise, a low-dose CT scan without application of contrast medium was acquired for attenuation correction and anatomic localization of PET uptake. Prior to imaging, patients were asked to void their bladder. The PET/CT acquisition and image reconstruction were performed according to our clinically established PET protocols for [^68^Ga]Ga-based tracers 9.

### Clinical assessments

PET image analysis was done using a Siemens software research prototype (MICIIS; Siemens Medical Solutions USA, Inc., Knoxville, TN, USA). Lesions with a standard uptake value (SUV) peak higher than a liver-specific threshold were subjected to segmentation as previously described 5,10. Smaller lesions measuring less than 0.5 ml were excluded. Additional foci with lower SUV values were manually included when required, using a 50% isocontour approach. The anatomical location according to the miTNM framework was noted for each lesion 7. PSMAvol was measured as body weight-adjusted total- and organ-specific tumor volume, the response was calculated as the absolute difference between the timepoints. PSMAvol always refers to body weight adjusted measurements in this manuscript. RECIP 1.0 criteria were defined in accordance with its original publication and the PPP (PSMA Prostate Progression) score was adapted to include Prostate-Specific Antigen (PSA) increase as an additional criterion for progression in cases of either single lesion growth or total tumor volume increase with a constant lesion count 9, 10. PSA values were recorded selectively at the PET/CT time points, based on the clinical information and their responses (relative change) were considered a surrogate parameter for response. The PSMA organ score was computed following the previously published methodology and model weights 8. The D'amico risk classification was applied using the freely available evedencio online calculator in accordance with the original definition, 12.

### Statistical analysis

For all statistical computations, R statistical software in version 4.3.2 was used (R Foundation for Statistical Computing, Vienna, Austria, www.R-project.org). Survival analysis was conducted using the log-rank test, univariate, and multivariate Cox proportional-hazards regression, as well as Kaplan-Meier curves. In all evaluations, two-tailed p-values that were ≤0.05 were regarded as statistically significant. For Cox regressions RECIP was numerically adjusted, while PSMAvol and its response were applied as continuous parameters without further stratification. The graphic abstract was partially designed using BioRender.com.

## Results

### Patient characteristics

Detailed patient characteristics are shown in *Table 1*. Median PSA level at baseline was 2.1 ng/ml, the ISUP grade was assessable in 33/73 patients (median 4). The median time between baseline and follow-up PET was 379 days (range: 76-1309 d). A total number of 26 patients died during follow-up. Both patients with HSPC (n = 42) and CRPC (n = 25), as well as 6 patients at primary staging (5 out of 6 with metastatic diseases and one patient without RP (radical prostatectomy) between the scans) were included. The classification of primary staging, HSPC, or CRPC was recorded according to the referrer's notification which was in accordance with EAU guidelines 13. Patients who started androgen deprivation therapy (ADT) for the first time less than 1 month before PET imaging were considered hormone sensitive even if the PET indication was re-staging for progression.

### Treatment between baseline and follow-up PET

For all patients included (main and further analyses), the treatments initiated after baseline PET are listed in *Table 2*. Briefly, a total of 21 localized therapies (including salvage surgery and radiotherapy (RT) and 30 systemic therapies (such as ADT, chemotherapy (CT) and Xofigo (Ra-223-dichlorid) were administered. For 27 patients, no prostate cancer specific therapy was reported.

### Baseline predictors of OS

In the present patient cohort, weight-adjusted total tumor volume was a statistically significant prognosticator of OS (mean 0.15, SD 0.51, HR 3.92, 95% CI 2.13 - 7.22, p < 0.001; n = 73). Likewise, the presence of a very high tumor burden, defined as 5 or more metastases was associated with shorter OS (HR 5.19, 95% CI 2.15 - 12.52, p < 0.001; n = 73). Castration resistance was a significant negative prognosticator in the total cohort (HR 4.79, 95% CI 2.14 - 10.76, p < 0.001; n = 73). Conversely, PSA levels (HR 1.00, p = 0.13; n = 64/73) reached no statistical significance at baseline.

### PSMAvol response, PPP and RECIP for OS Prediction

PSMAvol response (mean 0.05, SD: 0.22; n = 73) was significantly associated with OS (HR 10.48; 95% CI 2.05 - 53.56, p <0.005; n =73) as was the PPP response (HR 2.19, 95% CI 1.01 - 4.74, p < 0.05; n = 73). However, the RECIP assessment (PD vs. non-PD) was not statistically associated with OS (HR 1.26, p = 0.52; n = 73). Details are shown in *Table 3.*

### Subgroup of patients with HSPC at first PSMA-PET

Baseline PSMA tumor volume was significantly associated with OS in patients with HSPC (HR 3.75x10^5, 95% CI 2×10^2 - 7.05×10^8, p < 0.001; n = 42). While total PSMAvol response only showed a negative predictive tendency (HR 81.86, p = 0.12; n = 42), patients with poor PSMAvol response (upper quartile) had shorter OS compared to those with better response (p = 0.04; n =42). RECIP ([PD vs non-PD] HR 1.00, p = 0.96; n = 42) was not statistically significant. Details are shown in* Table 4.*

### Subgroup of patients with CRPC at first PSMA-PET

Tumor volume consistently emerged as a reliable predictor of OS, including in the CRPC cohort at baseline (HR 2.87, 95% CI 1.43 - 5.77, p < 0.005; n = 25) and in terms of response (HR 13.31, 95% CI 1.95 - 90.82, p < 0.01; n = 25). RECIP almost achieved a significant predictive value ([PD vs. non-PD] HR 2.72, p = 0.06; n = 25). Details are shown in* Table 4.*

### PSMAvol separately for the miTNM regions for response assessment

**Figure 3:**
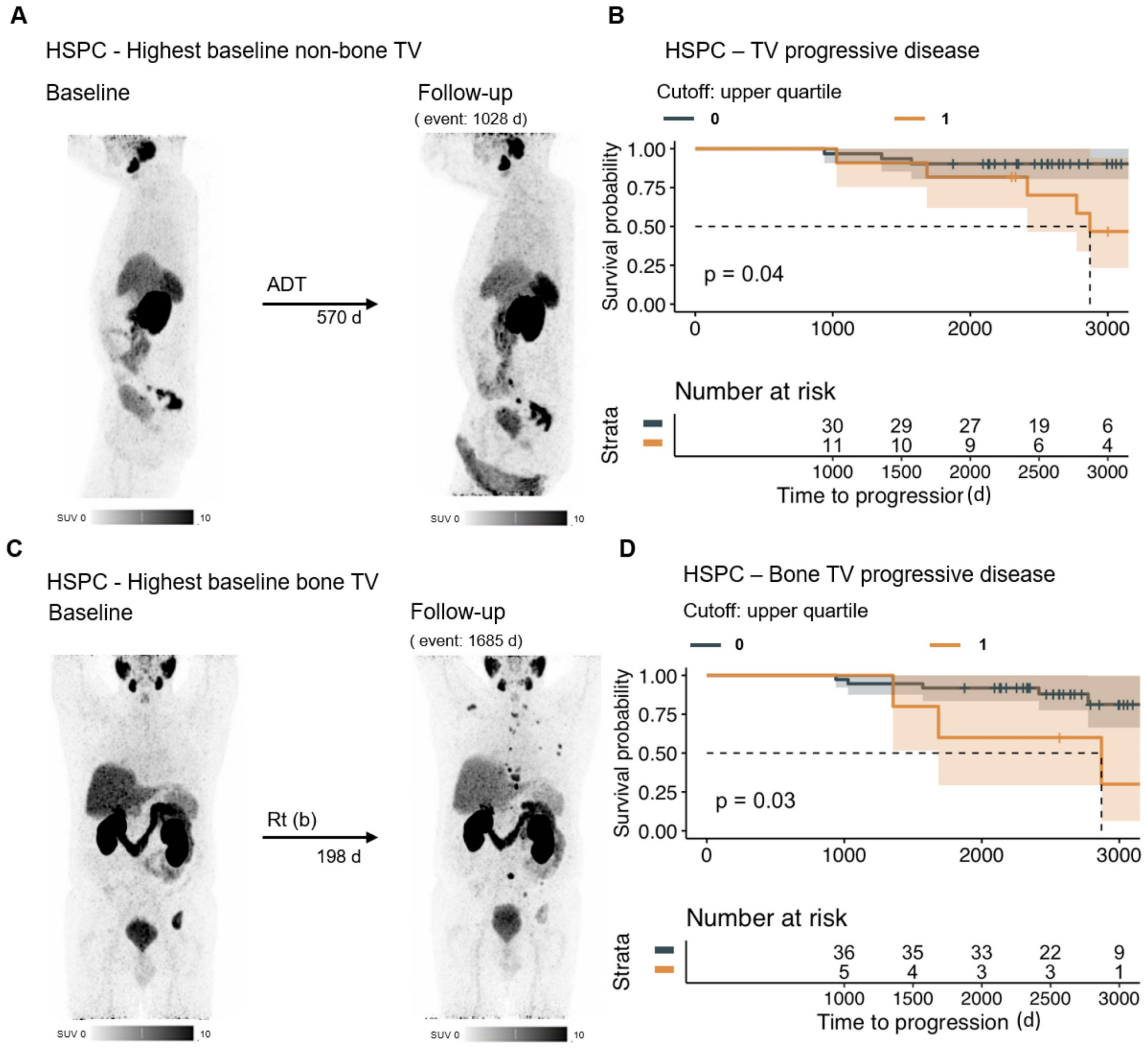
** PSMAvol response in HSPC.** The MIP of the baseline and follow-up PET of the patient with the highest non-bone PSMAvol of the HSPC group (at baseline) is shown (A). Patients with poor PSMAvol response (upper quartile change) in the HSPC group (n = 42) have a shorter overall survival time (B). To assess the relevance of bone PSMAvol in the HSPC (at baseline) group, the patient with the highest baseline bone PSMAvol is shown (C). Patients with a poor bone PSMAvol response (upper quartile change) in the HSPC group had shorter overall survival time (D). HSPC = hormone sensitive prostate cancer; TV = tumor volume; ADT = androgen deprivation therapy; Rt (b) = radiation therapy (bone).

Response in bone tumor volume was significantly associated with OS (HR 31.11, 95% CI 5.62 - 172.10, p < 0.001; n = 73), even when adjusted for patient age as covariate. Also, response in visceral metastases volume is significantly associated with OS in multivariate regression. Details are shown in* Table 5* and* Figure 3.* In the multivariate regression of HSPC patients, there was a significant impact observed on overall survival for distant lymph node metastases volume response (HR 1.97×10^4, 95% CI 8.04 - 4.84×10^7, p < 0.05; n =42), bone metastases volume response (HR 2.55×10^3; 95% CI 5.61 - 1.16×1^6, p < 0.05; n = 42), and other distant metastases volume response (HR 3.71×10-6, 95% CI 4.90×10^-10 - 2.81×10^-2, p < 0.01; n = 42).

## Discussion

At present, PSMA-PET is routinely used for the staging of patients with newly diagnosed intermediate to high-risk prostate cancer, patients with biochemically recurrent prostate cancer, or to assess eligibility of PSMA RLT (radioligand therapy) in castration resistant prostate cancer 14,15. However, the role of PSMA-PET to assess response to therapy, especially in earlier disease stages, is poorly elucidated to date. Here, it was shown that higher response in body weight adjusted PSMAvol is associated with improved OS in patients who were routinely staged with PSMA-PET and have not received PSMA RLT therapy. In this heterogeneously treated cohort, insufficient PSA decline or PSA progression was the main reason for the clinical initiation of follow-up PET.

Our data indicates that PSMA-PET is not only useful for disease localization to enable targeted therapy, but that PSMA-PET tumor volume is a biomarker that might be suited to quantitatively assess tumor burden and response to therapy. This is in line with findings by Kind *et al.*, showing that PSMA-volume is a prognostic biomarker for overall survival before PSMA-RLT and is useful as a quantitative response measure at the end of treatment 16. Also, in a subgroup of patients who were still hormone sensitive at baseline staging, response in local lymph node- and bone metastases tumor volume were associated with OS in the present analysis. Shiota *et al.* previously demonstrated that a substantial metastatic burden at the HSPC stage is a strong predictor of overall survival, without using PSMA-PET 17. However, despite early findings that tumor volume and high-grade tumor volume are key indicators for biochemical recurrence of HSPC patients following initial radical prostatectomy and the routinely use of high-volume tumor assessment to guide systemic therapy decisions, the critical role of PSMA-PET tumor volume within this disease stage has long been not at the center of interest, only to regain importance at the CRPC stage 18-20.

In our dataset, only PSA values at time of PSMA-PET imaging have been available. Changes in these values, without recognizing PSA changes in the interval between the PETs were not prognostic (data not shown). This contrasts with data from registration trials in mHSPC where deep PSA responses are known to be a strong prognostic indicator of overall survival 21,22. The lack of PSA levels is a limitation of the present study, a detailed follow-up of them might as well have been prognostic.

The recently published PROMISE V2.0 framework proposed organ specific tumor volume to assess the patient risk and measure response to therapy, as different locations of metastases have distinct effects on the OS risk 23. In the present heterogenous cohort, miTNM compliant PSMAvol responses were investigated. In the total cohort, bone tumor volume was the most relevant volumetric parameter associated with OS. This observation is supported by a work of Schmidkonz *et al.* who could show the utility of [^68^Ga]Ga-PSMA-11 PET/CT-derived quantitative volumetric tumor parameters for classification and determination of response to therapy of bone metastases in comparison with fully diagnostic conventional CT in patients with metastasized prostate cancer 24. Unlike our results, they observed a significant correlation with PSA trends, likely due to their access to detailed patient data throughout the therapy*.* This indicates the relevance of PSMA-PET for response assessment, which seems to be effective also in early stages of prostate cancer not only to localize disease, but also to assess the patient's risk 25,26. In this regard, PSMA-PET appears to be well-suited for assessing response to systemic non-RLT therapies, including chemotherapy and hormone therapy 27-29.

RECIP 1.0 was designed for late stage mCRPC patients and describes a novel system to assess response/progression to predict OS 30. Interestingly, RECIP response was not significantly associated with OS in the investigated patient cohort. This might be a limitation of the present study, as the cohort could have been too heterogeneous to objectify the prognostic value. However, the present work still strongly supports the use of PSMAvol as biomarker to measure response to therapy, which supports the rational of RECIP. Also, RECIP was almost significant in the CRPC cohort in this analysis. It could be that response assessment frameworks may not be applicable to all stages of prostate cancer, as in earlier disease stages the progression of individual sub-miTNM volumes may already have prognostic value without the total tumor volume showing significant progression that is needed for RECIP PD. Also, for example, a volumetric increase in local lymph nodes that can be irradiated could have a neglectable impact on overall survival compared with a bone metastasis with the same relative volumetric increase. Therefore, it could be inappropriate to not distinguish between the miTNM region of disease progression. In the present analysis, the impact of bone tumor volume was identified as the most significant factor affecting overall survival (OS) in the total cohort. Conversely for patients with HSPC at baseline, the PSMAvol response of distant lymph node metastases was the key determinant, suggesting the need for more nuanced evaluation criteria across different stages of prostate cancer. The assistance of machine learning for tumor volume delineation has improved the feasibility of total tumor volume segmentations 5,10. This advancement makes PSMAvol an easily accessible and straightforward feature for response assessment in PSMA-PET.

This study faces several limitations. It was conducted retrospectively and is therefore prone to a recall bias. Also, clinical data could not be retrieved for all patients, for example ISUP grading was accessible for too few patients to allow its inclusion in the survival analysis. Additionally, our cohort is notably diverse, particularly in terms of prior treatments. Given that the cohort was recruited from routine clinical practice, this heterogeneity appears inevitable. This diversity is representative of the everyday patient population, underscoring the need for radiological parameters to be universally applicable without constraints to maintain practical validity. Still, more data from more homogenous cohorts is needed to establish response assessment frameworks for specific disease stages. Overall survival data was obtained from the registration office ensuring high data quality. The included cohort of patients received baseline and follow up PETs on clinical indication, which might cause a selection bias as patients with non-suspicious findings were possibly not referred to follow-up PSMA-PET. Furthermore, the interval between the two PET scans cannot be standardized as most patients do not visit our clinic at regular intervals. Therefore, we did not examine the ratio of the follow-up PSA value to the baseline value, as continuous laboratory data were not available. For the same reason, no conclusion can be drawn that supports the routine use of PSMA-PET to monitor response of patients. However, the significant association of PSMAvol response with OS indicates that PSMAvol is feasible to assess the risk of patients and warrants the evaluation of PSMAvol for response assessment in clinical trials.

## Conclusion

In the total cohort, PSMAvol and response in PSMAvol were significantly associated with OS. In patients with hormone sensitive disease at the time of initial PSMA-PET, PSMAvol and organ-specific response were associated with OS. This indicates that PSMAvol might be a valuable parameter to monitor disease response and decide on treatment intensification, in addition to localizing the disease. Further studies in homogenous patient cohorts are needed to integrate PSMA-PET in clinical trials for quantitative response assessment and to compare the diagnostic performance of this approach with conventional imaging or PSA monitoring.

## Figures and Tables

**Figure 1:**
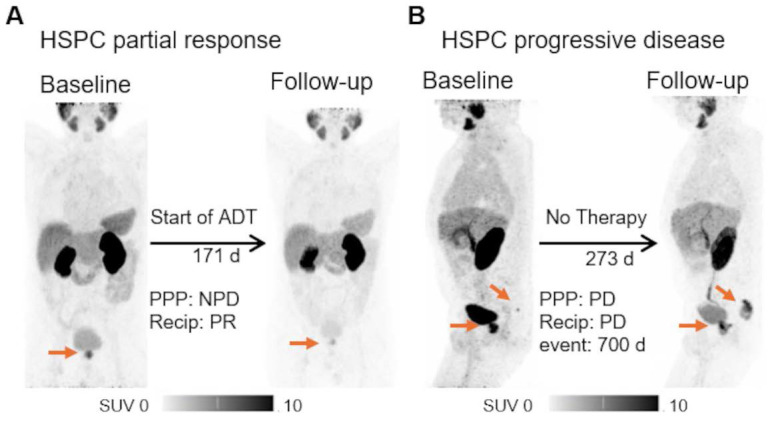
** Exemplary cases of PSMA-PET.** The patient underwent [^68^Ga]Ga-PSMA-11 PET for initial staging, with a high-risk D'Amico classification and PSA level of 56.90 ng/ml. Body weight adjusted local tumor volume of 0.072 ml/kg bodyweight was measured. A second PET scan post-ADT initiation revealed tumor shrinkage to 0.025/kg bodyweight ml, with corresponding drop in PSA to 7.0 ng/ml (A). A different patient experienced biochemical recurrence (PSA of 5.16 ng/ml) post radiotherapy. The initial [^68^Ga]Ga-PSMA-11 PET scan showed a weight-adjusted whole body tumor volume of 0.035 ml/kg bodyweight, indicating a local recurrence and a single bone metastasis. A follow-up scan during a watch and wait period at a PSA of 25.0 ng/ml demonstrated growth of both lesions, with a total body volume of 1.269 ml/kg bodyweight (B). Both patients were alive at the end of the survey period. HSPC = hormone sensitive prostate cancer; PPP = PSMA-PET progression; NPD = no progressive disease (does not fulfil the criteria for progression according to PPP); ADT = androgen deprivation therapy.

**Figure 2:**
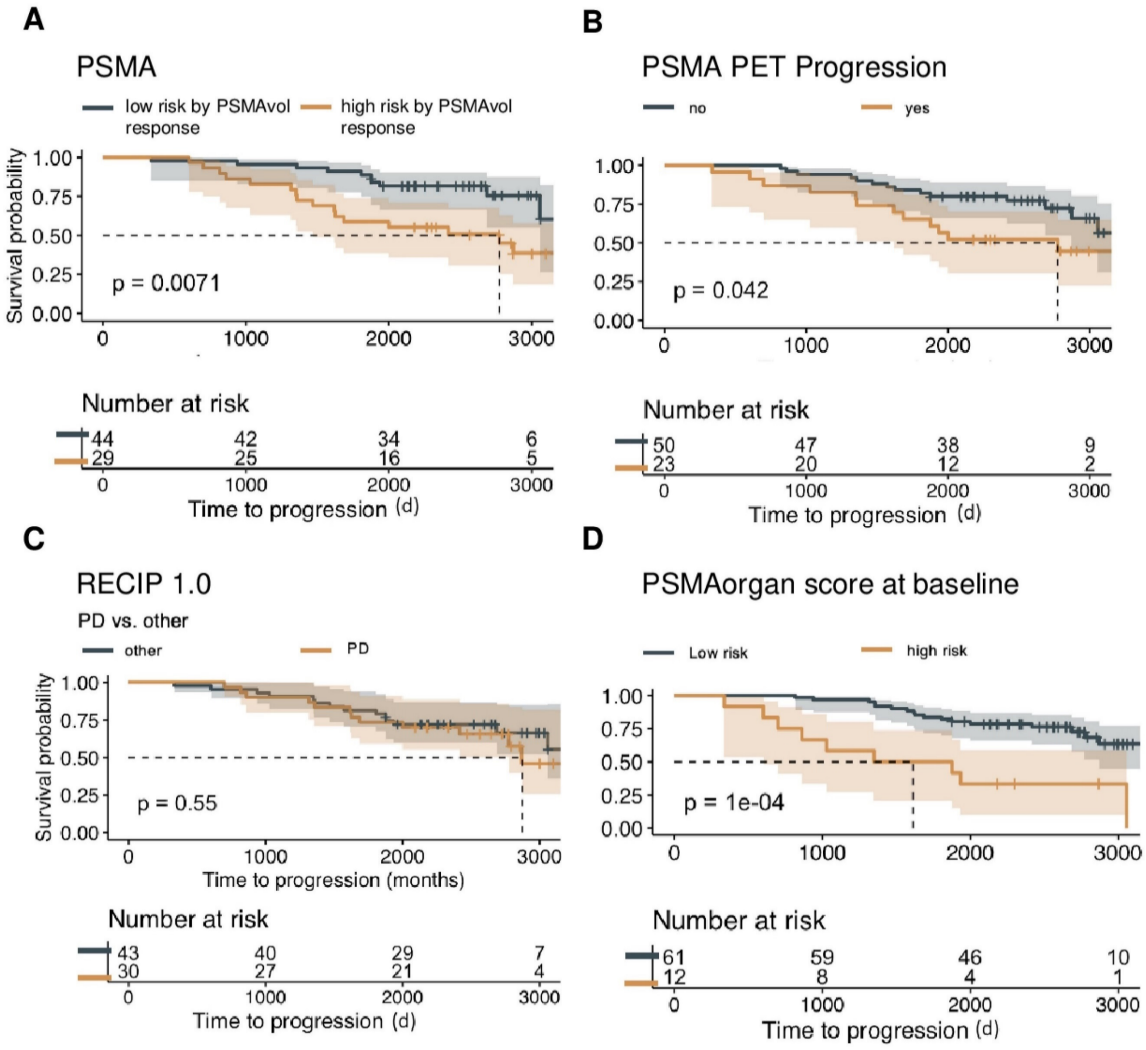
** Baseline PSMA-PET characteristics and OS.** Overall survival is shown in the non-Lu-PSMA cohort (n = 73) of patients, assessing the predictive value of continuous whole-body volume response (A), progression in the PSMA-PET progression score (B), RECIP 1.0 PD versus other responses (SD, PR, CR) (C), and the utilization of PSMA organ risk score calculated at the time of initial imaging with respect to patient survival (D), visualized by Kaplan-Meier curves. To determine an optimal cut point for the PSMA organ score and PSAM Vol, the MStats R (https://CRAN.R-project.org/package=mStats) package was utilized.

**Table 1:** Patient characteristics

	Patient groups			
Patient characteristics	Initial Staging therapienaiv	HSPC	CRPC	Overall
Patients	6 (8.2 %)	42 (56 %)	25 (33 %)	73
Age (years)	63 (50 -70)	67 (51-82)	71 (55-83)	68 (50-83)
PSA at baseline imaging (ng/ml)	43.0 (4.6-900.0)	1.2 (0.24-24.6)	5.2 (0.13-233.0)	2.1 (0.13-900.0)
ISUP				
1-3	0 (0 %)	11(26 %)	4 (16 %)	15 (21 %)
4	3 (38 %)	1 (2 %)	3 (12 %)	7 (9 %)
5	1 (17)	5 (12 %)	5 (20 %)	11 (15 %)
D'Amico Risk Classification				
Low Risk	0 (0 %)	0 (0)	0 (0)	0 (0)
Intermediate Risk	0 (0 %)	1 (2 %)	1 (4 %)	2 (3 %)
High Risk	4 (66 %)	13 (32 %)	11 (44 %)	28 (38 %)
Prior baseline treatments				
AS	0 (0 %)	1 (2 %)	0 (0 %)	1 (1 %)
RP	0 (0 %)	39 (93 %)	17 (68 %)	56 (75 %)
RT	0 (0 %)	24 (57 %)	19 (76 %)	43 (57 %)
SBRT	0 (0 %)	1 (2 %)	0 (0 %)	1 (1 %)
ADT	0 (0 %)	2 (5 %)	24 (96 %)	26 (35 %)
Abiraterone	0 (0 %)	0 (0 %)	3 (12 %)	3 (4 %)
Enzalutamide	0 (0 %)	0 (0 %)	2 (8 %)	2 (3 %)
Orchiectomy	0 (0%)	0 (0%)	2 (8 %)	2 (3 %)
Chemotherapy	0 (0 %)	0 (0 %)	3 (12 %)	3 (4 %)
PARP Inhibitor	0 (0 %)	0 (0 %)	0 (0 %)	0 (0 %)
LuPSMA	0 (0 %)	0 (0 %)	0 (0 %)	0 (0 %)
Xofigo	0 (0 %)	0 (0 %)	1 (5 %)	1 (1 %)
Days between baseline and follow up PET	308 (76-815)	389 (76-1233)	411 (126-1166)	379 (76-1309)

Abbreviations: PSA = prostate-specific antigen; ISUP = International Society of Urological Pathology; AS = active surveillance; RP = radical prostatectomy; RT = radiation therapy; SBRT = stereotactic body radiation therapy; ADT = androgen deprivation therapy. Qualitative data are number and percentage; continuous data are median and range.

**Table 2:** Treatment initiated after baseline PSMA-PET

Follow-up PET indications	*N*	RP	Surgery	RT	ADT	CT	Radium 223	None **
Continuously progressive PSA	41	0	2 LNE	8 (2 bone)	8 (4 *)	1	0	23
New onset of progressive PSA	7	0	3 LNE, 1 LR	3 (2 bone)	2 *.	0	0	0
PSA stable or declining	14	0	2 LNE	0	10 (6 *)	0	0	3
Individual request	11	0	1 LNE, 1 pul	0	4 (1*)	4 (1 *)	1	1

n = 73 Abbreviations: PSA = prostate-specific antigen; RP = radical prostatectomy; RT = radiation therapy; ADT = androgen deprivation therapy; CT = chemotherapy; LNE = lymph node excision; 1 LR = local recurrence resection, pul. = pulmonary; * = continuation of a therapy, already started before the baseline PET; ** none reported

**Table 3:** Association of OS with baseline characteristics and response parameters

Variable	Hazard Ratio		95% Confidence Interval		P-value
**Univariate Baseline** n = 73					
PSA (n = 64)	1.002		0.999 - 1.004		0.129
PSMAvol	3.920		2.127 - 7.224		< 0.001 (***)
very high tumor burden (≥ 5 metastases)	5.191		2.152 - 12.520		< 0.001 (***)
PSMA organ score	44.316		7.105 - 276.400		< 0.001 (***)
CRPC	4.794		2.137 - 10.760		< 0.001 (***)
**Multivariate Baseline** n = 73					
very high tumor burden (≥ 5 metastases)	5.022		1.688 - 14.940		0.004 (**)
PSMA organ score	4.490		0.546 - 36.940		0.162
CRPC	5.133		2.173 - 12.120		< 0.001 (***)
**Follow-up** n = 73					
PPP	2.186		1.009 - 4.737		0.047 (*)
PSMAvol response	10.481		2.051 - 53.560		0.005 (**)
RECIP PD vs. non-PD	1.264		0.584 - 2.763		0.522
**Multivariate Follow-up** n = 73					
PPP	1.685		0.726 - 3.909		0.224
PSMAvol response	6.502		1.018 - 41.552		0.048 (*)
RECIP PD vs. non-PD	1.084		0.492 - 2.382		0.849
					

Abbreviations: PSA = prostate-specific antigen; PSMAvol = PSMA Volume; CRPC = castration resistant prostate cancer; PPP = PSMA-PET progression; PD = progressive disease

**Table 4:** Association of OS with baseline characteristics and response metrics per disease group

Variable	Hazard Ratio	95% Confidence Interval	P-value
**HSPC univariate baseline** n = 42			
PSA (n = 36)	1.121	1.022 - 1.230	0.016 (*)
PSMAvol	3.755×10^5	2×10^2 - 7.052×10^8	≤0.001 (***)
very high tumor burden (≥ 5 metastases)	2.220	0.271 - 18.180	0.457
PSMA organ score	0.479	5.923×10^-5 - 3,867	0.872
**HSPC multivariate baseline**			
very high tumor burden (≥ 5 metastases)	2.660	0.443 - 15.970	0.285
PSMA organ score	1.789	0.001 - 6166.080	0.889
**HSPC response**			
PPP	2.058	0.487 - 8.694	0.326
PSMAvol response	81.861	0.318 - 2.107×10^4	0.120
RECIP PD vs. non-PD	0.996	0.248 - 4.00	0.995
**HSPC response multivariate**			
PPP	1.337	0.252 -7.078	0.733
PSMAvol response	98.752	0.076 - 128,300	0.209
RECIP PD vs. non-PD	0.658	0.134 - 3.228	0.606
**CRPC univariate baseline** n = 25			
PSA (n =22)	1.011	1.002 - 1.020	0.018 (*)
PSMAvol	2.874	1.432 - 5.766	0.003 (**)
very high tumor burden (≥ 5 metastases)	10.926	2.383 - 50.100	0.002 (**)
PSMA organ score	16.878	2.177 - 130.800	0.007 (**)
**CRPC multivariate baseline**			
very high tumor burden (≥ 5 metastases)	7.543	1.272 - 44.740	0.026 (*)
PSMA organ score	3.477	0.340 - 35.560	0.294
**CRPC response univariate**			
PPP	1.270	0.458 - 3.522	0.646
PSMAvol response	13.311	1.951 - 90.820	0.008 (**)
RECIP PD vs. non-PD	2.721	0.974 - 7.597	0.056
**CRPC response multivariate**			
PPP	0.929	0.319 -2.704	0.892
PSMAvol response	8.359	1.029 - 67.936	0.047
RECIP PD vs. non-PD	1.972	0.622 - 6.254	0.249

Abbreviations: HSPC = hormone sensitive prostate cancer; PSA = prostate-specific antigen; PSMAvol = PSMA Volume; PPP = PSMA-PET progression; PD = progressive disease; CRPC = castration resistant prostate cancer

**Table 5:** PSMAvol response shown separately by miTNM regions

miTNM			Univariate Cox regression of PSMAvol response		
(n = 73)	PSMAvol baseline	PSMAvol response	Hazard Ratio	95 % Confidence Interval	P-value
Prostate	0 (0 - 0.117)	±0 (- 0.078 - 0.113)	0.228	< 0.001 - 1.341e+09	0.897
Pelvic LN	0 (0 - 0.359)	±0 (-0.122 - 0.088)	0.017	< 0.001 - 31217	0.581
Distant LN	0 (0 - 0.251)	±0 (-0.153 - 0.288)	9.941	0.013 - 7830	0.500
Bones	0 (0 - 2.944)	±0 (-0.283 - 1.120)	31.106	5.623 - 172.100	< 0.001 (***)
Visceral	0 (0 - 0.783)	±0 (-0.298 - 0.461)	0.009	< 0.001 - 9.609	0.187
Qualitative data are median, (continuous data are range)					
			**Multivariate Cox regression of PSMAvol response**		
Prostate			0.082	< 0.001 - 330600	0.747
Pelvic LN			< 0.001	< 0.001 - 51.820	0.138
Distant LN			4.212	0.002 - 8591	0.711
Bones			37.270	5.460 - 254.400	< 0.001 (***)
Visceral			< 0.001	< 0.001 - 0.495	0.029 (*)
Age			1.058	0.999 - 1.120	0.054

Abbreviations: PSMAvol = PSMA Volume; LN = lymph nodes

## Abbreviations

ADT: androgen deprivation therapy
CI: confidence interval
CT: chemotherapy
CR: complete remission
CRPC: castration resistant prostate cancer
EANM: European Association of Nuclear Medicine
EAU: European Association of Urology
HR: hazard ratio
HSPC: hormone-sensitive prostate cancer
ISUP: International Society of Urological Pathology
LuPSMA: [^177^Lu]Lu-PSMA-617
mCRPC: metastatic castration-resistant prostate cancer
mHSPC: metastatic hormone-sensitive prostate cancer
miTNM: molecular imaging TNM system
n: number
OS: overall survival
p: probability-value
PCa: prostate cancer
PET: Positron Emission Tomography
PD: progressive disease
PPP: PSMA-PET Progression Criteria
PR: partial remission
PROMISE V2: Second Version of the Prostate Cancer Molecular Imaging Standardized Evaluation Framework Including Response Evaluation for Clinical Trials
PSA: Prostate-Specific Antigen
PSMA-PET: Prostate Specific Membrane Antigen Positron Emission Tomography
PSMA-PET/CT: Prostate Specific Membrane Antigen Positron Emission Tomography/Computer Tomography
PSMAvol: sum of PET volumes of all lesions
RECIP 1.0: Response Evaluation Criteria in PSMA-imaging v1.0
RLT: radioligand therapy
RP: radical prostatectomy
SD: standard deviation
SD (RECIP): stable disease
SUV: standard uptake value
TV: tumor volume
Xofigo: Ra-223-dichlorid
